# Comparison of Salivary TIMP-1 Levels in Periodontally Involved and Healthy Controls and the Response to Nonsurgical Periodontal Therapy

**DOI:** 10.1155/2014/363581

**Published:** 2014-01-09

**Authors:** Angel Fenol, Maya Rajan Peter, Jayachandran Perayil, Rajesh Vyloppillil, Anuradha Bhaskar

**Affiliations:** Department of Periodontics, Amrita School of Dentistry, Amrita Vishwa Vidyapeetham, Kochi 682041, India

## Abstract

*Background*. Periodontal disease is a chronic inflammatory condition affecting the supporting structures of the dentition. Periodontal destruction is an outcome of the imbalance between matrix metalloproteinases and tissue inhibitors of matrix metalloproteinases (TIMPs). We wanted to prove the hypothesis that salivary TIPM-1 level will vary in different people. A decrease in TIMP-1 level could make them more susceptible to periodontitis whereas a normal level could prevent increased tissue destruction thereby inhibiting the progression from gingivitis to periodontitis. This could probably pave the way for TIPM-1 to be a specific salivary biomarker and serve as a useful diagnostic and therapeutic tool in periodontitis. *Methods*. Whole unstimulated saliva of 2 ml was collected from twenty-five periodontally healthy and twenty-seven systemically healthy subjects with periodontitis. Clinical parameters recorded at baseline and reevaluated after four weeks in subjects with periodontitis following nonsurgical periodontal therapy were gingival index (GI), oral hygiene index-Simplified (OHI-S), probing pocket depth, and clinical attachment level (CAL). Salivary TIMP-1 levels in both were analyzed using a commercially available ELISA kit.

## 1. Introduction

Periodontal disease is a chronic disease of the oral cavity comprising a group of inflammatory conditions affecting the supporting structures of the dentition. Due to its importance in oral biofilm formation and host defence, secreted saliva may have a significant role in the establishment and progression of periodontal disease. Saliva, because of its locality, represents a key and very relevant diagnostic fluid for periodontal disease. Periodontal inflammatory mediators and tissue destructive molecules have been detected in the gingival tissues, gingival crevicular fluid, and saliva of patients affected by periodontitis [1].

Saliva contains biomarkers specific for the unique physiological aspects of periodontitis, and qualitative changes in the composition of these biomarkers could be diagnostic. Multiple contributing inflammatory mediators and tissue destructive molecules in this process have been shown to be correlated with disease onset and activity and could therefore be of diagnostic value as they have been detected in the saliva of patients affected by periodontitis [2]. Four distinct pathways may be involved in periodontal destruction: plasminogen dependent, phagocytic, osteoclastic pathway, and matrix metalloproteinase (MMP) pathway [3]. Experimental evidence suggests that the most important pathway involves Matrix metalloproteinases, as active collagenases and gelatinases which are found not only in the crevicular gingival fluid but also in saliva and in biopsy specimens of inflamed periodontal tissues [4].

Matrix mettalloproteinases (MMP's) form an important group of proteins that are involved not only in the degradation of matrix proteins in periodontitits but also in normal turn over in health and healing. It has been suggested that periodontal destruction is an outcome of the imbalance between matrix metalloproteinases and their inhibitors. Increase in matrix metalloproteinase and decrease in tissue inhibitors of matrix metalloproteinase levels initiate collagen degradation from connective tissue and alveolar bone. The levels of these specific salivary biomarkers could serve as a useful diagnostic and therapeutic tool in periodontitis.

The aims and objectives of this study were to (1) compare the salivary level of tissue inhibitor of matrix metalloproteinase (TIMP)-1 in periodontally involved subjects and healthy controls, (2) correlate the salivary TIMP-1 level of periodontally involved subjects with their clinical parameters of periodontitis before nonsurgical periodontal therapy, (3) correlate the salivary TIMP-1 level of periodontally involved subjects with their clinical parameters of periodontitis after nonsurgical periodontal therapy, and (4) compare the salivary TIMP-1 level in periodontally involved subjects, before and after nonsurgical periodontal therapy.

## 2. Materials and Methods

### 2.1. Subject Population and Study Design

The study population comprised 100 subjects in the age group of 35–55 years with a minimum number of 20 teeth, who had reported to the Department of Periodontics, Amrita School of Dentistry, Kochi. Twenty-five periodontally healthy subjects who had reported constituted the Control group which included systemically healthy subjects with probing pocket depth (PPD) ≤ 3 mm and with no clinical attachment loss. Of the 75 subjects who had periodontitis, 27 matched the criteria for study group. They were systemically healthy and had a probing depth of (PPD) ≥ 4-5 mm and clinical attachment level (CAL) ≥ 3-4 mm involving at least 7 teeth. Exclusion criteria included subjects with history of habits like usage of tobacco and/or consumption of alcohol, chronic inflammatory disorders of the skin and oral mucosa, known systemic illnesses; subjects on antibiotics and/or anti-inflammatory drugs within the last 6 months, subjects who underwent professional cleaning/periodontal treatment within the last 6 months, and pregnant women and lactating mothers.

The protocol was approved by the Ethical Committee of the Amrita School of Dentistry, Amrita Vishwa Vidyapeetham University, Kochi. The study was explained to all the subjects before their participation and a written informed consent was obtained. The individuals were divided into two groups, namely, the periodontally healthy control group (HC) and the periodontally diseased study group (PD).

### 2.2. Saliva Sampling

Whole unstimulated saliva of 2 mL was collected from the subjects [5] of both the groups in eppendorf tubes and stored at −80°C, which was subjected to further analysis in order to evaluate the presence of the biomarker TIMP-1. A detailed clinical examination of the selected diseased subjects with periodontitis was done. Standardization of the pocket depth was done using a custom made acrylic stent. The pocket depth was measured at six sites of each tooth (mesiobuccal, midbuccal, distobuccal, mesiolingual, midlingual, and distolingual). Clinical parameters analyzed were gingival index (GI) [6], oral hygiene index-Simplified (OHI-S) [7], probing pocket depth, and clinical attachment level (CAL). This was recorded as baseline. The subjects with periodontitis underwent supragingival scaling followed by subgingival scaling and root planning and the clinical parameters were reevaluated after four weeks [8–10]. The salivary TIMP-1 level was also reassessed at four weeks after nonsurgical periodontal therapy. Frozen saliva samples were thawed and centrifuged at 9,300 for 3 minutes prior to analysis. Salivary TIMP-1 and MMP-8 levels in both groups before nonsurgical periodontal therapy and in diseased group after nonsurgical periodontal therapy were analyzed using a commercially available ELISA kit (Quantikine Saliva Kit, R&D Systems Inc., USA) according to the manufacturer's instructions at the Department of Biochemistry, Christian Medical College Hospital And Research Centre, Vellore.

### 2.3. Statistical Analysis

The data collected were subjected to statistical analysis which included independent *t*-tests to evaluate the salivary TIMP-1 levels of periodontally healthy controls and periodontally diseased individuals, correlations to assess the association between the salivary TIMP-1 levels before and after nonsurgical periodontal therapy, and intra-group comparisons between the salivary TIMP-1 level and its correlations with their respective clinical parameters were also done, namely, the gingival index, clinical attachment level, and oral hygiene index.

## 3. Results

The purpose of the present study was to evaluate TIMP-1 as a salivary biomarker and its diagnostic significance in periodontal disease.

Table 1 shows a mean TIMP-1 value of 129.7 ± 53.4 in the healthy control group and 89.7 ± 43.0 in the periodontally diseased group. There is statistically significant correlation between the two groups (*P* < 0.05). Mean TIMP-1 value of 89.7 ± 43.0 before treatment and 80.7 ± 44.4 after nonsurgical periodontal therapy in the periodontally diseased group is also seen. There was no significant correlation between the two variables (*P* > 0.05).

Table 2 shows a mean gingival index (GI) score of 1.51 ± 0.19 before nonsurgical periodontal therapy and mean GI score of 0.75 ± 0.21 after nonsurgical periodontal therapy. There is statistically significant correlation between the two groups (*P* < 0.05). A mean CAL score of 5.08 ± 0.47 before nonsurgical periodontal therapy and mean CAL score of 4.24 ± 0.40 after nonsurgical periodontal therapy is seen. There is statistically significant correlation between the two groups (*P* < 0.05). Mean OHI score of 3.00 ± 0.51 before nonsurgical periodontal therapy and mean OHI score of 0.39 ± 0.23 after nonsurgical periodontal therapy were observed. There is statistically significant correlation between the two groups (*P* < 0.05).

Correlation coefficient between periodontally diseased TIMP-1 levels before nonsurgical periodontal therapy and gingival index score is seen to be 0.016 (Table 3). There is no significant correlation (*P* > 0.05). Correlation coefficient between periodontally diseased TIMP-1 levels before nonsurgical periodontal therapy and clinical attachment level is seen to be 0.169. There is no significant correlation (*P* > 0.05). Correlation coefficient between periodontally diseased TIMP-1 levels before nonsurgical periodontal therapy and oral hygiene index is seen to be −0.121. There is a negative correlation seen but it is not statistically significant (*P* > 0.05).

Correlation coefficient between periodontally diseased TIMP-1 levels after nonsurgical periodontal therapy and gingival index score is seen to be −0.35 (Table 4). There is a negative correlation but it is not statistically significant (*P* > 0.05). Correlation coefficient between periodontally diseased TIMP-1 levels after nonsurgical periodontal therapy and clinical attachment level is seen to be 0.068. There is no significant correlation (*P* > 0.05). Correlation coefficient between periodontally diseased TIMP-1 levels after nonsurgical periodontal therapy and oral hygiene index is seen to be −0.357. There is a negative correlation but it is not statistically significant (*P* > 0.05).

Mean MMP-8 value of 77.6 ± 25.3 was seen in the healthy control group and 94.3 ± 32.5 in the periodontally diseased group. There is statistically significant difference between the two groups (*P* < 0.05). Mean MMP-8 value of 94.3 ± 32.5 before treatment and 79.45 ± 30.1 after nonsurgical periodontal therapy in the periodontally diseased group is also seen. There was significant difference between the two groups (*P* < 0.05).

## 4. Discussion

Periodontal diseases are characterized by a loss of collagen fibres and other extracellular matrix constituents in periodontal tissues [11]. Matrix metalloproteinases (MMPs) are zinc-dependent endopeptidases that catalyze degradation of extracellular matrix proteins, thereby controlling such processes as development, tissue remodeling, wound healing, and tumor metastasis [12–14]. MMP-8, also called neutrophil collagenase-2, is one of the major collagenases that have a major part in the destruction of connective tissue and alveolar bone in periodontitis.

Periodontal health requires a balance between tissue destruction enzymes and their inhibitors. The activity of MMPs is controlled by regulation of expression and secretion, by proteolytic activation of proenzymes, and by the tissue inhibitors of metalloproteinases (TIMPs) [15, 16]. MMP-8 can be activated by other MMPs and inhibited by TIMP-1 and TIMP-2 [17]. TIMPs form 1 : 1, non-covalent complexes with MMPs, blocking access of substrates to the MMP catalytic site. TIMPs are highly specific for MMPs in general but not for any particular MMP. Functional specificity is conferred by other characteristics. TIMP-1 is an inducible protein, soluble and widely distributed. TIMP-1 is a 184 amino acid residue glycosylated protein, though glycosylation is not necessary for activity [18]. It has 12 cysteines (conserved among all TIMPs) that form disulfide bonds in a pattern that gives distinct N- and C-terminal domains [19]. The N-terminal domain contains sites that bind to the MMP substrate-binding site [20]. Binding of TIMP-1 does not leave a peptide bond in position for proteolysis and is not cleaved [16]. The TIMP/MMP complex can dissociate to yield enzyme and active TIMP-1 [21]. The C-terminal domain binds to an external site on MMPs, increasing overall affinity [22]. TIMP-1 binds with high affinity to the inactive pro-MMP-9, forming a complex in which TIMP-1 retains its ability to inhibit the activity of another active MMP via its N-terminal domain [23].

TIMP-1 is widely synthesized by many cells and tissues [15]. Transcription of the TIMP-1 gene is induced by pro-inflammatory cytokines (IL-1, IL-6, OSM, LIF, and TNF-*α*), TGF-*β*1, and phorbol esters [15, 24]. Many physiological functions of TIMP-1 are closely tied to the functions of MMPs, and an improper balance of MMP and TIMP production correlates with pathological conditions such as periodontitis, arthritis, tumor growth, and metastasis [15].

The primary objective of this study was to analyze the salivary TIMP-1 level in periodontally healthy controls and periodontally diseased subjects before and after nonsurgical periodontal therapy. The mean salivary TIMP-1 value of 129.7 ± 53.4 ng/mL was seen in the healthy control group and a mean salivary TIMP-1 value of 89.7 ± 43.0 ng/mL was seen in the periodontally diseased group. The salivary TIMP-1 level was seen to be significantly higher and showed statistical significance (*P* < 0.05) between the two groups (Table 1). This was in corroboration with an earlier study done by Hayakawa et al. in 1994 [25], where the salivary TIMP-1 levels were seen to be 273 ± 145 ng/mL in clinically healthy subjects in contrast to periodontally diseased subjects with TIMP-1 levels of 137 ± 67 ng/mL that were significantly lower. Other similar studies too have substantiated these findings [26, 27]. TIMPs are seen in various cells and tissues. MMP-8, also called neutrophil collagenase-2, is one of the important collagenases that have a major part in the destruction of connective tissue and alveolar bone in periodontitis. The activity of MMP is regulated by TIMP-1 by forming 1 : 1 noncovalent complexes with MMPs, blocking access of substrates to the MMP catalytic site. In healthy individuals, the TIMP-1 levels were found to be higher reflecting the capability of these individuals to counter the activity of MMPs as evidenced by healthy periodontium with normal clinical parameters. In patients who are prone to periodontal diseases, it was inferred that a greater amount of MMP activity was reflected through a decreased or less potent TIMP activity, as also evidenced by the destruction of periodontal tissues and altered clinical parameters. This has been further established in our results which have shown significantly higher TIMP-1 levels in the periodontally healthy group as opposed to that seen in the periodontally diseased group.

The salivary TIMP-1 levels of periodontally diseased subjects were evaluated 4 weeks after nonsurgical periodontal therapy. A mean TIMP-1 value of 89.7 ± 43.0 ng/mL before treatment and a mean TIMP-1 value of 80.7 ± 44.4 ng/mL after nonsurgical periodontal therapy in the periodontally diseased group were observed. There was no statistical significance between the two variables (*P* > 0.05) (Table 1). In this study, there was a decrease in the TIMP-1 level after 4 weeks of treatment. Previous studies [28] have shown that there is actually an increase in the TIMP-1 levels at 3 and 6 months after phase I therapy, which are contrary to our present finding. No studies have been conducted till date to assess the TIMP-1 levels after 4 weeks of treatment. The resultant decrease in TIMP-1 levels may be attributed to a fall in the MMPs immediately after nonsurgical periodontal therapy. This may have contributed to a decrease in the need for tissues to respond and could have led to a sudden decrease in the level of TIMP-1. The initial decrease in the TIMP-1 level will be reversed back to the genetically determined levels of TIMP-1, given an adequate time frame of 3 to 6 months. In the present study, TIMP-1 levels were analyzed after nonsurgical periodontal therapy in 4 weeks because it is the minimum time period required to assess the need for surgical therapy based on the tissue response to the phase I therapy. We wanted to evaluate whether there is a correlation between the tissue response and the TIMP-1 levels at 4 weeks after nonsurgical therapy and if TIMP-1 can be used as a suitable biomarker at this time to establish the need for surgical intervention. The TIMP-1 level was seen to decrease at 4 weeks in the study and showed a negative correlation between the clinical parameters. Hence it cannot be used as a marker at this stage. As it has been established through previous studies [28] that salivary TIMP-1 level increases 3 to 6 months after nonsurgical periodontal therapy and was positively correlated to the clinical parameters, it could be used as a marker to assess the periodontal status at that stage instead.

Further, the clinical parameters of periodontal disease were also analyzed. The parameters chosen in this study to reflect periodontal health were gingival index (GI), clinical attachment level (CAL), and oral hygiene index (OHI). They were assessed before and after 4 weeks of nonsurgical periodontal therapy. The gingival index score, clinical attachment level, and oral hygiene index scores showed statistically significant results following nonsurgical periodontal therapy. The mean gingival index (GI) score, CAL and OHI score were seen to be 1.51 ± 0.19, 5.08 ± 0.47 mm, and 3.00 ± 0.51, respectively, before nonsurgical periodontal therapy and mean values for gingival index (GI) score, CAL, and OHI score after nonsurgical periodontal therapy were found to be 0.75 ± 0.21, 4.24 ± 0.40 mm, and 0.39 ± 0.23, respectively. There was statistically significant correlation between the two groups in all the clinical parameters analyzed (*P* < 0.05) (Table 2). This further establishes the fact that nonsurgical periodontal therapy improves the clinical parameters like gingival index, clinical attachment level, and oral hygiene index.

We further correlated pretreatment TIMP-1 levels with the pretreatment clinical parameters and posttreatment TIMP-1 levels with posttreatment TIMP-1 levels. In the present study, the gingival index scores, clinical attachment level, and oral hygiene index scores had improved at 4 weeks after nonsurgical periodontal therapy. This highlights the benefits of nonsurgical periodontal therapy on the inflammatory process leading to a reduction in the MMP levels. Normally, the activation of MMPs is determined by the TIMP-1 levels, which, in this study, has been altered by the phase I therapy. Thus the effect of nonsurgical periodontal therapy at 4 weeks seems to have a greater influence on the MMP levels rather than TIMP-1/MMP interaction. This decreased interaction at 4 weeks corresponds to the reduction in the TIMP-1 level during the same period (Table 3).

A negative correlation between oral hygiene index and gingival index with TIMP-1 levels was established after 4 weeks of nonsurgical periodontal therapy. This suggests that an increase in the salivary TIMP-1 level might have led to the decrease in the gingival index score. An improvement in the oral hygiene index scores and hence a decrease in the pathogenic load will bring down the MMP levels. This can lead to a dip in the TIMP-1 values initially but will increase to the genetically predetermined levels (Table 4).

It can be thus inferred that TIMP-1 levels are found to be higher in periodontally healthy individuals than those who are prone to periodontitis. TIMP-1 level immediately after nonsurgical periodontal therapy were found to decrease as compared to their level prior to treatment at 4 weeks, even though studies have shown that their levels increase at 3, 4, and 6 months. The clinical parameters show a significant improvement after nonsurgical periodontal therapy. Gingival index score and oral hygiene index score show a negative correlation with TIMP-1 levels. It can be assumed therefore that TIMP-1 can be used as a marker to detect if the patient is prone to periodontal breakdown or if an inactive disease can become active in the near future. We can also infer that TIMP-1 is not a definitive marker and it cannot be correlated with clinical parameters at 4 weeks after periodontal therapy. But it can definitely be suggested as a reliable marker to assess the need for surgical phase and correlate with the clinical parameters after 3 months of nonsurgical therapy.

The limitation of the present study is that the period of study was for a short term, which may not provide the adequate duration required for various important clinical variations to reflect a conclusive difference. A longer period of assessment is therefore suggested to highlight the importance of the influence of these variations. A larger sample size if used for the study would have been optimal and more reflective of the parameters studied and could be applied more relevantly as the study pertained to the utility of a specific biomarker in periodontal disease assessment. The period of evaluation of TIMP-1 levels after nonsurgical periodontal therapy observed in this study was 4 weeks. A longer time period along with additional evaluation of salivary TIMP-1 levels in study subjects requiring surgical therapy may give better direction to changes in the TIMP-1 levels and provide significant correlation with clinical parameters. Thus the incorporation of a longer period of assessment after treatment and a study group involving surgical therapy is recommended. ELISA was the method used to evaluate salivary TIMP-1 levels in this study, with the intention to provide an economical alternative for periodontal disease assessment. However, the differentiation of periodontitis subjects from controls by the immunofluorometric assay (IFMA) technique has proved to be stronger than the widely used enzyme-linked immune sorbent assay (ELISA) method, based on salivary MMP-8 detection [27]. One explanation can be the difference in specificities between the IFMA and ELISA antibodies [29]. The antibody used in IFMA identifies neutrophilic and fibroblast-type MMP-8 isotypes and in particular their active forms [29]. Unlike IFMA, the ELISA method detects all forms of MMP-8, as the antibodies have been produced against full-size MMP-8 [30]. The ability of IFMA to detect both PMN-type and fibroblast-type MMP-8 and especially in their active forms, explains its strength in differentiating periodontitis and healthy subjects [27].

Mean MMP-8 value of 77.6 ± 25.3 was seen in the healthy control group and 94.3 ± 32.5 in the periodontally diseased group. There is statistically significant difference between the two groups (*P* < 0.05). Mean MMP-8 value of 94.3 ± 32.5 before treatment and 79.45 ± 30.1 after nonsurgical periodontal therapy in the periodontally diseased group is also seen. There was significant difference between the two groups (*P* < 0.05). This shows that the levels of MMP-8 released during periodontal infection can definitely be managed after periodontal treatment, thus reducing the amount of destruction that can occur, but the presence of an adequate amount of TIMP-1 levels in an individual can limit periodontal tissue destruction by limiting the MMP's that are produced by the host.

## 5. Conclusion

Based on the methodology used and on the results seen in the present study, it can be concluded that

1. the TIMP-1 levels are found to be higher in periodontally healthy individuals than periodontally diseased subjects. There is a statistical significant correlation between the two groups;
2. TIMP-1 level evaluated 4 weeks after nonsurgical periodontal therapy was found to decrease as compared to their level prior to treatment. There was no statistical significance in the two groups;
3. the clinical parameters show a significant improvement after nonsurgical periodontal therapy. There was statistical significance in all the clinical parameters, 4 weeks after nonsurgical periodontal therapy;
4. the mean gingival index score and oral hygiene index score showed a negative correlation with TIMP-1 levels. It can be assumed therefore that TIMP-1 can be used as a marker to detect if the patient is prone to periodontal breakdown or if an inactive disease can become active in the near future. We can also infer that TIMP-1 is not an ideal marker and it cannot be correlated with clinical parameters at 4 weeks after periodontal therapy; rather it can definitely be suggested as a reliable marker to assess the need for surgical phase and correlate with the clinical parameters after 3 months of nonsurgical therapy;
5. mean MMP-8 value of 77.6 ± 25.3 was seen in the healthy control group and 94.3 ± 32.5 in the periodontally diseased group. There is statistically significant difference between the two groups (*P* < 0.05). Mean MMP-8 value of 94.3 ± 32.5 before treatment and 79.45 ± 30.1 after nonsurgical periodontal therapy in the periodontally diseased group is also seen.

The results are interpreted in terms of scientific literature pertinent to each comparison, keeping in mind the limited study sample and the potential for measurement error in the subjective assessment of the TIMP-1 levels.

It can be summarized that salivary TIMP-1 can definitely be used as a biomarker in detecting and differentiating between periodontally healthy controls and periodontally involved subjects. Salivary TIMP-1 is not an ideal marker for assessment at 4 weeks after nonsurgical periodontal therapy and it does not correlate with the clinical parameters at 4 weeks after periodontal therapy. It can definitely be suggested as a reliable marker to assess the need for surgical phase and correlate with the clinical parameters after 3 months of nonsurgical therapy.

Within the discipline of Point-of-Care (POC) diagnostics, the use of saliva as the source of biomarkers is particularly appealing owing to its noninvasive collection and well tolerance by patients. Rigorous studies that result in improvements in our diagnostic capacity regarding these areas would greatly facilitate the clinician's treatment of periodontal diseases and the assessment of periodontal status in medical and research settings.

## Figures and Tables

**Table 1:** Correlation between TIMP-1 levels in healthy control group and periodontally diseased group before and after nonsurgical periodontal therapy.

	*N*	Mean TIMP-1	SD	*t*	*P*
Category					
Healthy control	25	129.7	53.4	2.987	0.004
Disease group	27	89.7	43.0		
Disease group					
Before	27	89.7	43.0	1.304	0.204
After	27	80.7	44.4		

*N* denotes the number of sample subjects, SD: standard deviation, *t*-student *t* test and *P* value-power of significance.

**Table 2:** Correlation of the mean gingival index scores, mean CAL scores, and mean OHI scores before and after nonsurgical periodontal therapy.

	*N*	Mean	SD	*t*	*P*
GI					
Pre	27	1.51	0.19	19.361	<0.001
Post	27	0.75	0.21		
CAL					
Pre	27	5.08	0.47	16.443	<0.001
Post	27	4.24	0.40		
OHI					
Pre	27	3.00	0.51	24.746	<0.001
Post	27	0.39	0.23		

*N* denotes the no. of sample subjects, SD: standard deviation, *t*-student *t* test and *P* value-power of significance.

**Table 3:** Correlation of periodontally diseased TIMP-1 levels with clinical parameters namely gingival index, clinical attachment level, and oral hygiene index before nonsurgical periodontal therapy.

PD TIMP-1	*r*	*P*
GI Pre	0.016	0.937
CAL Pre	0.169	0.398
OHI Pre	−0.121	0.549

PD: periodontally diseased group, *r*: correlation coefficient, *P*: power of significance.

**Table 4:** Correlation of periodontally diseased TIMP-1 levels with clinical parameters namely gingival index, clinical attachment level, and oral hygiene index one month after nonsurgical periodontal therapy.

T TIMP-1	*r*	*P*
GI 1	−0.350	0.074
CAL 1	0.068	0.736
OHI 1	−0.357	0.068

T: Treated subjects; *r*: correlation coefficient; *P*: power of significance.
